# Supplementary material for: Racial and Ethnic Disparities in Years of Potential Life Lost Attributable to COVID-19 in the United States: An Analysis of 45 States and the District of Columbia
Source: Int J Environ Res Public Health. 2021 Mar 12;18(6):2921. doi: 10.3390/ijerph18062921 (PMC8000338; doi:10.3390/ijerph18062921)
Supplement: Supplementary file 1 [file ijerph-18-02921-s001.zip › Supplementary Materials/Table S1.pdf]

State	NH White	NH Black	Hispanic	NH Asian	NH AIAN
US	[1,353,819 – 1,377,390]	[775,987 – 798,111]	[1,022,177 – 1,044,441]	[128,910 – 149,805]	[68,629 – 89,077]
AL	[12,949 – 14,528]	[14,595 – 14,991]	[1,461 – 1,580]	[69 – 108]	[35 – 71]
AZ	[10,192 – 12,752]	[1,543 – 2,205]	[22,176 – 23,145]	[495 – 1,122]	[11,318 – 12,136]
AR	[7,488 – 8,609]	[3,142 – 3,316]	[1,307 – 1,416]	[36 – 54]	[12 – 28]
CA	[21,213 – 21,950]	[10,197 – 10,756]	[99,053 – 100,254]	[10,366 – 10,915]	[366 – 673]
CO	[5,770 – 7,556]	[1,084 – 1,208]	[6,731 – 7,006]	[510 – 613]	[305 – 386]
CT	[6,831 – 8,202]	[3,567 – 3,823]	[3,427 – 3,658]	[275 – 380]	[0 – 61]
DE	[1,554 – 3,078]	[1,064 – 1,173]	[203 – 246]	[10 – 20]	[10 – 20]
DC	[281 – 834]	[4,287 – 4,493]	[1,937 – 2,056]	[78 – 101]	[10 – 20]
FL	[29,642 – 31,306]	[28,728 – 29,256]	[30,640 – 31,194]	[1,425 – 1,554]	[132 – 164]
GA	[17,140 – 18,586]	[26,052 – 26,563]	[7,388 – 7,629]	[678 – 774]	[30 – 40]
ID	[2,392 – 3,709]	[22 – 45]	[904 – 1,009]	[56 – 81]	[35 – 62]
IL	[20,147 – 22,899]	[18,922 – 19,553]	[25,097 – 25,735]	[2,152 – 2,505]	[36 – 264]
IN	[17,084 – 19,503]	[5,192 – 5,477]	[2,759 – 2,956]	[196 – 272]	[22 – 75]
IA	[9,235 – 10,388]	[794 – 876]	[1,185 – 1,289]	[126 – 174]	[79 – 101]
KS	[4,969 – 6,759]	[937 – 1,116]	[1,983 – 2,195]	[79 – 185]	[164 – 289]
KY	[8,828 – 10,170]	[1,716 – 1,872]	[1,077 – 1,166]	[69 – 97]	[0 – 18]
LA	[10,948 – 12,032]	[20,822 – 21,299]	[1,329 – 1,465]	[161 – 231]	[55 – 108]
MD	[6,218 – 7,490]	[12,981 – 13,365]	[7,827 – 8,087]	[639 – 760]	[23 – 61]
MA	[13,739 – 15,762]	[3,919 – 4,439]	[4,211 – 4,713]	[889 – 1,310]	[22 – 362]
MI	[21,204 – 22,771]	[20,378 – 21,232]	[2,958 – 3,515]	[245 – 718]	[132 – 583]
MN	[7,429 – 8,598]	[1,961 – 2,102]	[1,367 – 1,481]	[1,221 – 1,337]	[311 – 375]
MS	[8,499 – 9,493]	[14,968 – 15,369]	[324 – 401]	[88 – 158]	[1,346 – 1,469]
MO	[14,636 – 16,458]	[4,606 – 4,843]	[1,619 – 1,739]	[139 – 198]	[35 – 61]
MT	[1,852 – 2,651]	[55 – 75]	[105 – 144]	[32 – 48]	[1,577 – 1,700]
NE	[4,101 – 5,551]	[413 – 494]	[1,464 – 1,592]	[55 – 91]	[55 – 91]
NV	[4,195 – 5,523]	[2,084 – 2,235]	[7,731 – 7,987]	[1,325 – 1,466]	[150 – 191]
NH	[802 – 2,143]	[36 – 70]	[12 – 45]	[32 – 65]	[0 – 20]
NJ	[25,477 – 26,484]	[20,227 – 20,706]	[33,258 – 33,791]	[5,013 – 5,263]	[79 – 101]
NM	[1,242 – 2,532]	[69 – 91]	[4,434 – 4,644]	[12 – 28]	[7,232 – 7,464]
NY	[47,678 – 59,498]	[55,669 – 62,238]	[76,085 – 82,646]	[17,130 – 23,324]	[79 – 5,966]
NC	[5,979 – 7,035]	[6,641 – 6,944]	[4,017 – 4,244]	[190 – 285]	[79 – 167]
ND	[2,040 – 3,475]	[22 – 61]	[123 – 170]	[0 – 34]	[632 – 729]
OH	[20,730 – 21,718]	[7,029 – 7,302]	[1,175 – 1,279]	[329 – 394]	[36 – 55]
OK	[6,304 – 8,532]	[897 – 1,003]	[1,548 – 1,669]	[104 – 147]	[2,137 – 2,292]
OR	[2,119 – 2,609]	[113 – 144]	[1,640 – 1,761]	[81 – 126]	[69 – 98]
PA	[25,569 – 27,175]	[12,144 – 12,573]	[5,873 – 6,151]	[1,215 – 1,413]	[0 – 81]
RI	[2,134 – 3,260]	[465 – 569]	[615 – 740]	[113 – 174]	[0 – 48]
SC	[8,223 – 9,374]	[11,263 – 11,613]	[1,835 – 1,943]	[69 – 92]	[32 – 48]
SD	[2,403 – 3,233]	[56 – 75]	[166 – 194]	[80 – 101]	[1,150 – 1,259]
TN	[17,079 – 19,214]	[9,377 – 9,691]	[2,869 – 3,029]	[157 – 226]	[43 – 82]
TX	[38,200 – 42,254]	[22,343 – 22,944]	[125,304 – 126,456]	[2,512 – 2,816]	[350 – 547]
UT	[3,459 – 4,465]	[36 – 55]	[1,708 – 1,835]	[105 – 145]	[347 – 411]
VA	[7,303 – 8,791]	[7,364 – 7,683]	[4,482 – 4,710]	[584 – 721]	[12 – 77]
WA	[4,856 – 6,385]	[472 – 569]	[3,732 – 3,929]	[733 – 857]	[620 – 716]
WV	[2,257 – 3,078]	[88 – 131]	[0 – 10]	[20 – 30]	[10 – 20]
WI	[10,177 – 11,933]	[2,016 – 2,162]	[2,890 – 3,055]	[593 – 667]	[355 – 421]

Table S1: Conservative 95% interval estimates of total COVID-19-attributable YPLL before age 75 for NH Whites, NH Blacks, Hispanics, NH Asians, and NH AIAN's in the U.S. and in each examined state with respect to cumulative COVID-19 deaths according to data from the National Center for Health Statistics as of 30 December 2020.
