# Supplementary material for: Racial and Ethnic Disparities in Years of Potential Life Lost Attributable to COVID-19 in the United States: An Analysis of 45 States and the District of Columbia
Source: Int J Environ Res Public Health. 2021 Mar 12;18(6):2921. doi: 10.3390/ijerph18062921 (PMC8000338; doi:10.3390/ijerph18062921)
Supplement: Supplementary file 1 [file ijerph-18-02921-s001.zip › Supplementary Materials/Table S2.pdf]

State	NH White	NH Black	Hispanic	NH Asian	NH AIAN
US	[177,636 – 179,096]	[51,054 – 52,514]	[56,097 – 57,557]	[10,725 – 12,185]	[3,318 – 4,778]
AL	[3,427 – 3,470]	[1,654 – 1,689]	[103 – 152]	[6 – 55]	[4 – 53]
AZ	[3,283 – 3,410]	[209 – 344]	[2,270 – 2,389]	[105 – 240]	[884 – 1,019]
AR	[2,440 – 2,456]	[441 – 449]	[129 – 145]	[5 – 45]	[4 – 36]
CA	[6,865 – 6,909]	[1,471 – 1,531]	[10,418 – 10,478]	[2,519 – 2,579]	[81 – 149]
CO	[2,472 – 2,532]	[191 – 243]	[989 – 1,041]	[132 – 192]	[29 – 105]
CT	[3,738 – 3,797]	[731 – 790]	[483 – 542]	[62 – 121]	[2 – 61]
DE	[499 – 523]	[167 – 191]	[17 – 41]	[3 – 27]	[3 – 27]
DC	[105 – 121]	[610 – 626]	[123 – 147]	[6 – 34]	[1 – 9]
FL	[10,182 – 10,207]	[3,641 – 3,658]	[5,188 – 5,213]	[265 – 298]	[7 – 54]
GA	[4,561 – 4,586]	[3,239 – 3,264]	[541 – 574]	[128 – 168]	[3 – 36]
ID	[1,012 – 1,045]	[3 – 36]	[129 – 154]	[5 – 41]	[5 – 41]
IL	[8,327 – 8,426]	[2,585 – 2,668]	[2,415 – 2,506]	[510 – 609]	[4 – 103]
IN	[6,154 – 6,187]	[850 – 875]	[289 – 306]	[54 – 87]	[3 – 36]
IA	[3,588 – 3,604]	[101 – 125]	[117 – 133]	[46 – 70]	[6 – 54]
KS	[2,062 – 2,123]	[163 – 232]	[228 – 289]	[17 – 89]	[17 – 89]
KY	[2,635 – 2,660]	[317 – 342]	[69 – 94]	[6 – 39]	[2 – 27]
LA	[3,179 – 3,223]	[2,612 – 2,670]	[159 – 217]	[23 – 81]	[5 – 63]
MD	[2,688 – 2,726]	[2,162 – 2,200]	[579 – 617]	[205 – 243]	[4 – 42]
MA	[7,421 – 7,533]	[757 – 869]	[660 – 772]	[285 – 397]	[4 – 123]
MI	[6,725 – 6,842]	[2,808 – 2,933]	[323 – 448]	[82 – 223]	[32 – 173]
MN	[4,113 – 4,156]	[255 – 298]	[142 – 192]	[173 – 216]	[45 – 95]
MS	[2,281 – 2,307]	[1,817 – 1,851]	[22 – 63]	[16 – 57]	[86 – 127]
MO	[5,142 – 5,184]	[794 – 828]	[137 – 163]	[55 – 97]	[5 – 63]
MT	[716 – 732]	[4 – 25]	[15 – 36]	[4 – 25]	[185 – 201]
NE	[1,533 – 1,575]	[66 – 110]	[147 – 191]	[5 – 49]	[5 – 49]
NV	[1,173 – 1,197]	[272 – 288]	[725 – 733]	[290 – 298]	[16 – 64]
NH	[598 – 628]	[5 – 35]	[4 – 34]	[4 – 34]	[0 – 9]
NJ	[9,060 – 9,077]	[2,843 – 2,860]	[3,456 – 3,481]	[985 – 1,016]	[6 – 37]
NM	[513 – 544]	[6 – 37]	[642 – 659]	[4 – 35]	[640 – 657]
NY	[15,459 – 16,449]	[8,147 – 9,113]	[8,581 – 9,531]	[2,631 – 3,597]	[20 – 1,010]
NC	[1,964 – 1,999]	[1,045 – 1,072]	[289 – 328]	[45 – 84]	[26 – 65]
ND	[981 – 1,029]	[3 – 51]	[7 – 55]	[3 – 51]	[70 – 118]
OH	[7,526 – 7,543]	[1,242 – 1,275]	[144 – 169]	[86 – 119]	[4 – 38]
OK	[2,035 – 2,067]	[162 – 178]	[162 – 186]	[24 – 56]	[287 – 295]
OR	[841 – 867]	[7 – 52]	[158 – 184]	[43 – 77]	[6 – 51]
PA	[11,049 – 11,102]	[2,127 – 2,180]	[658 – 711]	[288 – 341]	[3 – 56]
RI	[1,172 – 1,197]	[81 – 114]	[115 – 152]	[7 – 44]	[2 – 27]
SC	[2,749 – 2,774]	[1,675 – 1,700]	[117 – 142]	[15 – 64]	[4 – 45]
SD	[1,140 – 1,156]	[4 – 36]	[8 – 42]	[6 – 40]	[142 – 166]
TN	[4,505 – 4,539]	[1,138 – 1,164]	[200 – 234]	[28 – 62]	[3 – 37]
TX	[9,682 – 9,760]	[2,699 – 2,777]	[13,441 – 13,519]	[486 – 557]	[41 – 119]
UT	[901 – 917]	[5 – 45]	[205 – 229]	[27 – 59]	[36 – 76]
VA	[3,001 – 3,040]	[1,236 – 1,275]	[410 – 449]	[182 – 221]	[4 – 43]
WA	[2,120 – 2,165]	[89 – 134]	[404 – 447]	[203 – 248]	[74 – 117]
WV	[738 – 755]	[18 – 35]	[2 – 19]	[2 – 19]	[2 – 19]
WI	[4,297 – 4,321]	[342 – 374]	[290 – 306]	[76 – 92]	[57 – 89]

Table S2: Intervals denoting the entire plausible range of total COVID-19 deaths for NH Whites, NH Blacks, Hispanics, NH Asians, and NH AIAN's in the U.S. and in each examined state with respect to cumulative COVID-19 deaths according to data from the National Center for Health Statistics as of 30 December 2020.
