# Supplementary material for: Racial and Ethnic Disparities in Years of Potential Life Lost Attributable to COVID-19 in the United States: An Analysis of 45 States and the District of Columbia
Source: Int J Environ Res Public Health. 2021 Mar 12;18(6):2921. doi: 10.3390/ijerph18062921 (PMC8000338; doi:10.3390/ijerph18062921)
Supplement: Supplementary file 1 [file ijerph-18-02921-s001.zip › Supplementary Materials/Table S3.pdf]

State	NH White		NH Black		Hispanic		NH Asian		NH AIAN	
	% YPLL	% Deaths	% YPLL	% Deaths	% YPLL	% Deaths	% YPLL	% Deaths	% YPLL	% Deaths
US	[40.1 – 40.8%]	[58.9 – 59.4%]	[23.0 – 23.6%]	[16.9 – 17.4%]	[30.3 – 31.0%]	[18.6 – 19.1%]	[3.8 – 4.4%]	[3.6 – 4.0%]	[2.0 – 2.6%]	[1.1 – 1.6%]
AL	[41.0 – 47.0%]	[65.3 – 66.1%]	[45.6 – 51.3%]	[31.5 – 32.2%]	[4.6 – 9.5%]	[2.0 – 2.9%]	[0.21 – 2.9%]	[0.11 – 1.0%]	[0.11 – 1.9%]	[0.08 – 1.0%]
AZ	[19.9 – 25.9%]	[47.2 – 49.0%]	[3.0 – 8.4%]	[3.0 – 4.9%]	[43.2 – 49.9%]	[32.6 – 34.3%]	[0.97 – 5.7%]	[1.5 – 3.4%]	[22.2 – 29.2%]	[12.7 – 14.6%]
AR	[48.2 – 61.6%]	[77.7 – 78.2%]	[19.7 – 27.3%]	[14.0 – 14.3%]	[8.3 – 15.0%]	[4.1 – 4.6%]	[0.22 – 3.2%]	[0.16 – 1.4%]	[0.08 – 1.5%]	[0.13 – 1.1%]
CA	[14.4 – 15.1%]	[31.7 – 31.9%]	[6.9 – 8.1%]	[6.8 – 7.1%]	[67.5 – 69.5%]	[48.1 – 48.4%]	[7.1 – 8.2%]	[11.6 – 11.9%]	[0.25 – 1.0%]	[0.37 – 0.69%]
CO	[31.6 – 45.6%]	[63.4 – 64.9%]	[5.8 – 13.2%]	[4.9 – 6.2%]	[36.3 – 49.8%]	[25.4 – 26.7%]	[2.8 – 11.1%]	[3.4 – 4.9%]	[1.6 – 8.8%]	[0.74 – 2.7%]
CT	[41.4 – 52.3%]	[73.6 – 74.8%]	[22.0 – 33.3%]	[14.4 – 15.6%]	[20.9 – 30.7%]	[9.5 – 10.7%]	[1.7 – 8.0%]	[1.2 – 2.4%]	[0.00 – 3.2%]	[0.04 – 1.2%]
DE	[37.0 – 69.4%]	[69.8 – 73.1%]	[24.1 – 54.9%]	[23.4 – 26.7%]	[4.6 – 32.8%]	[2.4 – 5.7%]	[0.23 – 4.8%]	[0.42 – 3.8%]	[0.23 – 4.8%]	[0.42 – 3.8%]
DC	[3.4 – 11.4%]	[12.0 – 13.8%]	[52.3 – 68.9%]	[69.6 – 71.4%]	[23.9 – 38.7%]	[14.0 – 16.8%]	[0.94 – 10.9%]	[0.68 – 3.9%]	[0.12 – 2.2%]	[0.11 – 1.0%]
FL	[31.5 – 33.6%]	[52.5 – 52.6%]	[30.4 – 32.2%]	[18.8 – 18.9%]	[32.5 – 34.6%]	[26.8 – 26.9%]	[1.5 – 2.9%]	[1.4 – 1.5%]	[0.14 – 1.5%]	[0.04 – 0.28%]
GA	[31.4 – 35.0%]	[53.5 – 53.8%]	[47.8 – 52.0%]	[38.0 – 38.3%]	[13.5 – 17.1%]	[6.4 – 6.7%]	[1.2 – 4.1%]	[1.5 – 2.0%]	[0.06 – 0.64%]	[0.04 – 0.42%]
ID	[49.5 – 77.1%]	[84.8 – 87.5%]	[0.43 – 8.8%]	[0.25 – 3.0%]	[17.9 – 39.4%]	[10.8 – 12.9%]	[1.1 – 15.8%]	[0.42 – 3.4%]	[0.67 – 11.9%]	[0.42 – 3.4%]
IL	[28.6 – 32.8%]	[59.6 – 60.4%]	[26.8 – 30.1%]	[18.5 – 19.1%]	[35.5 – 39.5%]	[17.3 – 18.0%]	[3.0 – 6.1%]	[3.7 – 4.4%]	[0.05 – 1.8%]	[0.03 – 0.74%]
IN	[62.5 – 69.7%]	[83.2 – 83.7%]	[18.6 – 24.3%]	[11.5 – 11.8%]	[9.8 – 13.5%]	[3.9 – 4.1%]	[0.69 – 4.1%]	[0.73 – 1.2%]	[0.08 – 2.3%]	[0.04 – 0.49%]
IA	[62.7 – 80.1%]	[91.5 – 91.9%]	[5.3 – 15.0%]	[2.6 – 3.2%]	[7.9 – 15.2%]	[3.0 – 3.4%]	[0.84 – 6.2%]	[1.2 – 1.8%]	[0.53 – 6.6%]	[0.15 – 1.4%]
KS	[43.1 – 66.9%]	[80.4 – 82.7%]	[8.1 – 27.7%]	[6.4 – 9.0%]	[17.1 – 36.4%]	[8.9 – 11.3%]	[0.67 – 15.9%]	[0.66 – 3.5%]	[1.4 – 19.5%]	[0.66 – 3.5%]
KY	[67.0 – 77.4%]	[86.0 – 86.8%]	[12.6 – 20.1%]	[10.3 – 11.2%]	[7.9 – 12.7%]	[2.3 – 3.1%]	[0.50 – 6.0%]	[0.20 – 1.3%]	[0.00 – 0.95%]	[0.07 – 0.88%]
LA	[29.7 – 34.7%]	[52.6 – 53.4%]	[57.4 – 64.6%]	[43.2 – 44.2%]	[3.6 – 9.4%]	[2.6 – 3.6%]	[0.43 – 2.1%]	[0.38 – 1.3%]	[0.15 – 2.6%]	[0.08 – 1.0%]
MD	[20.5 – 25.5%]	[47.3 – 48.0%]	[43.2 – 49.3%]	[38.1 – 38.7%]	[26.0 – 31.5%]	[10.2 – 10.9%]	[2.1 – 5.9%]	[3.6 – 4.3%]	[0.07 – 1.7%]	[0.07 – 0.74%]
MA	[54.0 – 62.7%]	[80.1 – 81.3%]	[15.4 – 22.9%]	[8.2 – 9.4%]	[16.5 – 24.0%]	[7.1 – 8.3%]	[3.5 – 9.8%]	[3.1 – 4.3%]	[0.09 – 4.5%]	[0.04 – 1.3%]
MI	[43.9 – 48.6%]	[66.4 – 67.6%]	[42.4 – 47.4%]	[27.7 – 29.0%]	[6.1 – 9.6%]	[3.2 – 4.4%]	[0.51 – 4.0%]	[0.81 – 2.2%]	[0.27 – 3.1%]	[0.32 – 1.7%]
MN	[49.1 – 62.8%]	[86.0 – 86.9%]	[12.9 – 21.7%]	[5.3 – 6.2%]	[9.1 – 22.2%]	[3.0 – 4.0%]	[8.0 – 15.1%]	[3.6 – 4.5%]	[2.0 – 9.3%]	[0.94 – 2.0%]
MS	[30.7 – 35.9%]	[53.4 – 54.1%]	[54.4 – 61.2%]	[42.6 – 43.4%]	[1.2 – 5.6%]	[0.52 – 1.5%]	[0.32 – 2.9%]	[0.37 – 1.3%]	[4.8 – 9.3%]	[2.0 – 3.0%]
MO	[61.7 – 71.0%]	[82.9 – 83.6%]	[19.1 – 25.3%]	[12.8 – 13.4%]	[6.6 – 9.7%]	[2.2 – 2.6%]	[0.57 – 3.8%]	[0.89 – 1.6%]	[0.14 – 2.2%]	[0.08 – 1.0%]
MT	[40.3 – 58.6%]	[75.5 – 77.2%]	[1.2 – 13.8%]	[0.42 – 2.6%]	[2.2 – 16.2%]	[1.6 – 3.8%]	[0.68 – 9.2%]	[0.42 – 2.6%]	[34.1 – 52.3%]	[19.5 – 21.2%]
NE	[48.3 – 72.5%]	[84.9 – 87.3%]	[4.8 – 24.6%]	[3.7 – 6.1%]	[17.6 – 41.1%]	[8.1 – 10.6%]	[0.63 – 10.7%]	[0.28 – 2.7%]	[0.64 – 10.8%]	[0.28 – 2.7%]
NV	[22.6 – 31.8%]	[46.2 – 47.2%]	[11.2 – 17.1%]	[10.7 – 11.4%]	[41.7 – 50.8%]	[28.6 – 28.9%]	[7.1 – 10.6%]	[11.4 – 11.7%]	[0.79 – 6.8%]	[0.63 – 2.5%]
NH	[52.5 – 95.9%]	[93.1 – 97.8%]	[1.6 – 38.6%]	[0.78 – 5.5%]	[0.57 – 27.1%]	[0.62 – 5.3%]	[1.5 – 36.9%]	[0.62 – 5.3%]	[0.00 – 13.8%]	[0.00 – 1.4%]
NJ	[29.3 – 30.8%]	[55.1 – 55.2%]	[23.2 – 24.6%]	[17.3 – 17.4%]	[38.3 – 40.2%]	[21.0 – 21.2%]	[5.8 – 7.8%]	[6.0 – 6.2%]	[0.09 – 0.97%]	[0.04 – 0.23%]
NM	[8.4 – 17.4%]	[27.9 – 29.5%]	[0.45 – 5.3%]	[0.33 – 2.0%]	[29.8 – 36.7%]	[34.9 – 35.8%]	[0.08 – 1.5%]	[0.22 – 1.9%]	[48.5 – 57.0%]	[34.8 – 35.7%]
NY	[22.8 – 28.4%]	[43.1 – 45.9%]	[26.5 – 31.7%]	[22.7 – 25.4%]	[36.2 – 41.2%]	[23.9 – 26.6%]	[8.1 – 12.9%]	[7.3 – 10.0%]	[0.04 – 4.0%]	[0.06 – 2.8%]
NC	[31.4 – 38.6%]	[57.5 – 58.6%]	[34.6 – 40.9%]	[30.6 – 31.4%]	[21.3 – 29.0%]	[8.5 – 9.6%]	[1.0 – 8.4%]	[1.3 – 2.5%]	[0.42 – 4.7%]	[0.76 – 1.9%]
ND	[43.2 – 80.1%]	[87.9 – 92.2%]	[0.44 – 14.0%]	[0.27 – 4.6%]	[2.5 – 32.6%]	[0.63 – 4.9%]	[0.01 – 7.8%]	[0.27 – 4.6%]	[12.6 – 40.0%]	[6.3 – 10.6%]
OH	[65.9 – 71.1%]	[83.2 – 83.4%]	[22.3 – 27.6%]	[13.7 – 14.1%]	[3.7 – 6.3%]	[1.6 – 1.9%]	[1.0 – 4.0%]	[0.95 – 1.3%]	[0.11 – 1.4%]	[0.04 – 0.42%]
OK	[43.9 – 61.5%]	[73.0 – 74.1%]	[6.0 – 12.9%]	[5.8 – 6.4%]	[10.4 – 21.1%]	[5.8 – 6.7%]	[0.70 – 6.0%]	[0.86 – 2.0%]	[14.3 – 20.9%]	[10.3 – 10.6%]
OR	[35.5 – 56.1%]	[75.9 – 78.2%]	[1.9 – 22.4%]	[0.63 – 4.7%]	[27.6 – 46.0%]	[14.3 – 16.6%]	[1.4 – 12.2%]	[3.9 – 6.9%]	[1.1 – 16.1%]	[0.54 – 4.6%]
PA	[53.6 – 58.2%]	[77.9 – 78.3%]	[25.4 – 29.6%]	[15.0 – 15.4%]	[12.2 – 15.3%]	[4.6 – 5.0%]	[2.5 – 5.5%]	[2.0 – 2.4%]	[0.00 – 1.4%]	[0.02 – 0.39%]
RI	[40.4 – 71.4%]	[82.7 – 84.4%]	[8.8 – 36.2%]	[5.7 – 8.0%]	[12.2 – 44.6%]	[8.1 – 10.7%]	[2.1 – 29.0%]	[0.49 – 3.1%]	[0.00 – 9.8%]	[0.14 – 1.9%]
SC	[33.9 – 41.1%]	[59.6 – 60.1%]	[46.8 – 54.9%]	[36.3 – 36.8%]	[7.5 – 10.4%]	[2.5 – 3.1%]	[0.28 – 3.4%]	[0.32 – 1.4%]	[0.13 – 1.7%]	[0.09 – 0.97%]
SD	[42.5 – 67.9%]	[85.3 – 86.5%]	[0.95 – 13.4%]	[0.30 – 2.7%]	[3.0 – 28.5%]	[0.60 – 3.1%]	[1.4 – 17.6%]	[0.45 – 3.0%]	[20.4 – 45.5%]	[10.6 – 12.4%]
TN	[53.4 – 60.1%]	[76.2 – 76.7%]	[28.9 – 33.4%]	[19.2 – 19.7%]	[8.9 – 14.7%]	[3.4 – 4.0%]	[0.49 – 3.9%]	[0.47 – 1.0%]	[0.13 – 2.2%]	[0.05 – 0.63%]
TX	[19.7 – 21.8%]	[36.6 – 36.9%]	[11.5 – 13.0%]	[10.2 – 10.5%]	[64.5 – 66.8%]	[50.8 – 51.1%]	[1.3 – 2.2%]	[1.8 – 2.1%]	[0.18 – 1.3%]	[0.16 – 0.45%]
UT	[36.1 – 63.2%]	[70.0 – 71.2%]	[0.35 – 6.6%]	[0.39 – 3.5%]	[17.9 – 40.2%]	[15.9 – 17.8%]	[1.1 – 11.2%]	[2.1 – 4.6%]	[3.5 – 18.1%]	[2.8 – 5.9%]
VA	[33.6 – 40.9%]	[61.5 – 62.3%]	[33.7 – 40.2%]	[25.3 – 26.1%]	[20.6 – 27.4%]	[8.4 – 9.2%]	[2.7 – 7.3%]	[3.7 – 4.5%]	[0.06 – 2.8%]	[0.08 – 0.88%]
WA	[35.3 – 49.3%]	[70.5 – 72.0%]	[3.4 – 13.1%]	[3.0 – 4.5%]	[27.3 – 39.7%]	[13.4 – 14.9%]	[5.3 – 15.8%]	[6.8 – 8.3%]	[4.4 – 12.3%]	[2.5 – 3.9%]
WV	[79.8 – 95.8%]	[94.7 – 96.9%]	[2.8 – 16.4%]	[2.3 – 4.5%]	[0.01 – 2.4%]	[0.26 – 2.4%]	[0.64 – 9.0%]	[0.26 – 2.4%]	[0.33 – 5.8%]	[0.26 – 2.4%]
WI	[55.3 – 66.3%]	[84.2 – 84.7%]	[10.9 – 21.2%]	[6.7 – 7.3%]	[15.5 – 22.3%]	[5.7 – 6.0%]	[3.1 – 7.7%]	[1.5 – 1.8%]	[1.9 – 7.4%]	[1.1 – 1.7%]

Table S3: Conservative 95% interval estimates of the percentage of total COVID-19-attributable YPLL before age 75 and intervals denoting the entire plausible range of the percentage of total COVID-19 deaths for NH Whites, NH Blacks, Hispanics, NH Asians, and NH AIAN's in the U.S. and in each examined state with respect to cumulative COVID-19 deaths according to data from the National Center for Health Statistics as of 30 December 2020.
