# Supplementary material for: Racial and Ethnic Disparities in Years of Potential Life Lost Attributable to COVID-19 in the United States: An Analysis of 45 States and the District of Columbia
Source: Int J Environ Res Public Health. 2021 Mar 12;18(6):2921. doi: 10.3390/ijerph18062921 (PMC8000338; doi:10.3390/ijerph18062921)
Supplement: Supplementary file 1 [file ijerph-18-02921-s001.zip › Supplementary Materials/Table S4.pdf]

State	NH White YPLL	NH White Mortality	NH Black YPLL	NH Black Mortality	Hispanic YPLL	Hispanic Mortality	NH Asian YPLL	NH Asian Mortality	NH AIAN YPLL	NH AIAN Mortality
US	[63.3 – 64.5]	[7.4 – 7.4]	[182.7 – 188.0]	[14.2 – 14.7]	[186.7 – 191.2]	[14.5 – 15.0]	[63.8 – 74.2]	[6.2 – 7.0]	[244.0 – 321.0]	[13.5 – 19.9]
AL	[36.6 – 41.8]	[8.9 – 9.0]	[115.8 – 122.7]	[15.7 – 16.1]	[108.7 – 169.3]	[11.0 – 17.4]	[8.3 – 109.4]	[1.1 – 13.9]	[10.2 – 169.2]	[1.2 – 19.4]
AZ	[22.0 – 28.9]	[5.4 – 5.7]	[52.8 – 122.0]	[10.0 – 15.6]	[126.5 – 137.6]	[19.6 – 20.6]	[21.0 – 95.4]	[5.5 – 11.4]	[423.3 – 524.4]	[42.4 – 48.8]
AR	[31.8 – 37.1]	[9.1 – 9.2]	[73.7 – 84.8]	[13.1 – 13.4]	[103.0 – 135.2]	[16.4 – 17.5]	[7.7 – 92.1]	[1.5 – 14.8]	[4.7 – 78.8]	[1.7 – 17.3]
CA	[12.6 – 13.0]	[3.4 – 3.4]	[42.7 – 49.1]	[7.4 – 7.7]	[79.9 – 81.4]	[12.3 – 12.4]	[16.1 – 19.0]	[4.1 – 4.2]	[17.2 – 71.9]	[4.3 – 8.0]
CO	[13.4 – 18.2]	[6.2 – 6.4]	[50.5 – 88.3]	[13.0 – 16.3]	[76.3 – 86.8]	[17.6 – 18.5]	[30.5 – 89.2]	[10.2 – 14.1]	[73.3 – 304.2]	[8.0 – 36.2]
CT	[23.2 – 29.3]	[10.5 – 10.8]	[102.5 – 149.0]	[26.6 – 28.8]	[80.7 – 105.5]	[17.9 – 20.1]	[20.3 – 72.0]	[6.3 – 11.8]	[0.47 – 555.6]	[2.3 – 78.5]
DE	[22.4 – 53.4]	[5.9 – 6.4]	[49.1 – 100.2]	[10.0 – 11.1]	[27.8 – 140.2]	[4.1 – 12.3]	[3.0 – 45.9]	[1.1 – 10.4]	[18.8 – 320.9]	[5.1 – 61.7]
DC	[15.8 – 36.6]	[5.5 – 6.3]	[140.6 – 171.5]	[19.7 – 20.3]	[298.7 – 394.1]	[27.5 – 33.5]	[22.6 – 262.2]	[3.0 – 26.0]	[42.6 – 963.9]	[4.1 – 58.4]
FL	[21.9 – 23.6]	[5.1 – 5.2]	[91.6 – 94.7]	[14.4 – 14.5]	[59.2 – 62.0]	[11.4 – 11.5]	[20.9 – 38.5]	[4.7 – 5.3]	[21.5 – 227.6]	[1.1 – 9.1]
GA	[28.1 – 30.8]	[7.4 – 7.5]	[81.4 – 85.7]	[14.4 – 14.6]	[101.5 – 114.8]	[13.6 – 14.4]	[17.8 – 48.8]	[4.8 – 6.0]	[10.4 – 132.4]	[1.2 – 16.3]
ID	[15.4 – 24.7]	[6.6 – 6.9]	[21.3 – 347.9]	[5.9 – 83.1]	[71.2 – 110.1]	[16.8 – 19.2]	[15.7 – 197.2]	[2.0 – 20.9]	[16.9 – 231.8]	[2.7 – 25.7]
IL	[23.0 – 26.6]	[8.4 – 8.6]	[109.1 – 119.0]	[17.2 – 17.7]	[148.0 – 158.3]	[22.7 – 23.6]	[31.5 – 56.5]	[9.1 – 10.7]	[13.0 – 472.3]	[1.5 – 44.6]
IN	[30.0 – 34.6]	[10.4 – 10.5]	[92.7 – 111.3]	[19.8 – 20.3]	[96.7 – 115.2]	[15.8 – 16.5]	[16.5 – 61.8]	[8.5 – 11.0]	[10.4 – 338.0]	[1.6 – 21.0]
IA	[32.0 – 36.5]	[10.5 – 10.6]	[94.4 – 167.3]	[21.0 – 23.2]	[115.0 – 153.4]	[17.7 – 19.2]	[24.2 – 109.6]	[15.3 – 19.4]	[71.9 – 817.1]	[7.7 – 84.2]
KS	[21.1 – 29.4]	[7.6 – 7.9]	[61.5 – 162.8]	[14.3 – 19.1]	[89.3 – 141.5]	[17.8 – 21.3]	[7.8 – 165.9]	[3.3 – 16.5]	[56.9 – 687.9]	[8.1 – 52.2]
KY	[21.6 – 25.2]	[6.5 – 6.6]	[51.7 – 73.6]	[12.2 – 13.2]	[90.7 – 126.8]	[8.6 – 13.5]	[9.0 – 103.1]	[1.3 – 11.7]	[0.21 – 91.1]	[1.7 – 26.7]
LA	[37.3 – 41.3]	[10.3 – 10.4]	[150.5 – 164.3]	[22.9 – 23.4]	[76.1 – 149.5]	[11.4 – 15.1]	[20.9 – 84.7]	[3.7 – 13.6]	[17.7 – 287.4]	[1.7 – 24.1]
MD	[17.9 – 22.2]	[6.8 – 6.9]	[70.0 – 78.3]	[14.9 – 15.2]	[157.6 – 179.2]	[19.8 – 21.9]	[16.7 – 41.2]	[6.2 – 7.4]	[10.3 – 251.6]	[2.2 – 27.4]
MA	[23.9 – 28.0]	[11.4 – 11.6]	[83.7 – 115.9]	[21.7 – 24.8]	[73.8 – 96.4]	[18.5 – 21.4]	[22.9 – 51.2]	[10.0 – 13.4]	[12.9 – 731.3]	[2.7 – 90.7]
MI	[25.1 – 27.1]	[7.4 – 7.6]	[154.9 – 168.6]	[24.7 – 25.8]	[82.7 – 119.0]	[14.1 – 19.9]	[9.2 – 59.2]	[4.2 – 10.6]	[19.2 – 206.7]	[5.7 – 33.0]
MN	[14.8 – 17.5]	[7.5 – 7.6]	[76.6 – 100.2]	[18.3 – 21.6]	[79.1 – 129.5]	[15.3 – 20.5]	[66.2 – 91.2]	[13.3 – 16.4]	[56.8 – 206.5]	[11.3 – 27.6]
MS	[47.3 – 53.2]	[11.3 – 11.5]	[142.8 – 155.8]	[22.0 – 22.4]	[57.1 – 180.6]	[4.3 – 16.1]	[30.2 – 210.1]	[6.4 – 26.2]	[942.4 – 1,636.5]	[67.8 – 106.9]
MO	[27.5 – 31.4]	[8.9 – 9.0]	[71.6 – 85.2]	[15.0 – 15.6]	[87.6 – 110.9]	[11.2 – 13.9]	[12.7 – 64.3]	[7.4 – 11.7]	[9.8 – 140.1]	[1.6 – 21.9]
MT	[17.9 – 27.0]	[6.4 – 6.6]	[85.8 – 998.4]	[7.5 – 171.0]	[40.0 – 222.0]	[7.6 – 22.6]	[24.0 – 320.6]	[4.8 – 41.0]	[294.7 – 411.5]	[44.1 – 47.7]
NE	[25.6 – 35.3]	[8.4 – 8.7]	[52.9 – 190.8]	[13.1 – 19.7]	[116.1 – 193.8]	[20.7 – 25.3]	[10.1 – 164.6]	[1.7 – 22.8]	[34.4 – 520.3]	[5.1 – 64.0]
NV	[22.8 – 33.0]	[6.0 – 6.3]	[80.9 – 103.9]	[12.8 – 13.4]	[111.3 – 118.8]	[17.5 – 17.7]	[40.6 – 52.5]	[10.1 – 10.5]	[47.5 – 367.2]	[5.5 – 23.9]
NH	[5.5 – 17.3]	[4.1 – 4.4]	[19.4 – 307.5]	[4.7 – 45.4]	[5.1 – 124.8]	[1.9 – 18.6]	[9.1 – 146.9]	[1.7 – 17.1]	[0.00 – 335.6]	[0.00 – 20.4]
NJ	[45.1 – 47.3]	[13.2 – 13.2]	[173.8 – 181.1]	[29.2 – 29.4]	[208.6 – 216.2]	[31.2 – 31.5]	[61.0 – 81.1]	[15.0 – 15.5]	[46.1 – 497.9]	[3.4 – 23.9]
NM	[13.7 – 34.9]	[4.1 – 4.6]	[16.1 – 171.3]	[1.6 – 12.7]	[49.0 – 54.7]	[8.3 – 8.5]	[3.9 – 61.4]	[1.3 – 13.4]	[406.8 – 439.5]	[45.4 – 46.9]
NY	[39.9 – 51.1]	[10.6 – 11.4]	[195.4 – 230.6]	[31.5 – 35.3]	[232.2 – 259.7]	[33.5 – 37.4]	[96.9 – 152.2]	[17.7 – 24.2]	[12.1 – 1,198.2]	[3.3 – 163.0]
NC	[8.0 – 9.5]	[2.5 – 2.5]	[29.7 – 32.6]	[5.8 – 6.0]	[61.4 – 77.9]	[7.7 – 8.7]	[6.5 – 43.6]	[2.8 – 4.5]	[6.5 – 67.9]	[2.5 – 6.4]
ND	[31.5 – 54.4]	[12.5 – 13.4]	[15.9 – 501.0]	[6.2 – 170.8]	[49.8 – 702.8]	[7.0 – 127.4]	[0.38 – 486.8]	[7.0 – 171.0]	[219.6 – 471.7]	[34.0 – 61.8]
OH	[20.4 – 21.4]	[6.8 – 6.9]	[51.7 – 60.7]	[10.6 – 10.9]	[41.3 – 57.1]	[7.2 – 8.2]	[14.1 – 39.4]	[5.4 – 6.8]	[9.9 – 127.3]	[1.2 – 14.1]
OK	[21.8 – 30.6]	[6.3 – 6.4]	[34.0 – 56.0]	[8.0 – 8.6]	[64.1 – 89.7]	[11.4 – 12.2]	[12.1 – 74.3]	[4.2 – 9.0]	[68.0 – 81.9]	[11.8 – 12.1]
OR	[5.8 – 7.2]	[2.1 – 2.2]	[10.9 – 116.0]	[1.0 – 10.7]	[45.9 – 57.0]	[7.8 – 9.3]	[3.9 – 24.9]	[2.9 – 5.0]	[11.9 – 140.5]	[1.3 – 12.8]
PA	[22.8 – 24.5]	[8.2 – 8.2]	[94.8 – 106.1]	[19.3 – 19.8]	[84.7 – 98.9]	[16.2 – 17.5]	[31.5 – 56.4]	[10.2 – 11.9]	[0.10 – 268.5]	[1.3 – 27.1]
RI	[23.1 – 39.3]	[10.7 – 11.1]	[79.9 – 241.7]	[18.0 – 23.2]	[59.7 – 146.5]	[16.8 – 19.3]	[25.2 – 302.6]	[2.4 – 19.7]	[0.83 – 828.5]	[4.3 – 78.2]
SC	[21.6 – 25.3]	[6.9 – 7.0]	[83.9 – 93.1]	[14.8 – 15.1]	[77.2 – 93.4]	[8.1 – 11.2]	[6.4 – 71.1]	[2.3 – 10.3]	[15.3 – 197.6]	[2.2 – 28.7]
SD	[29.7 – 42.2]	[12.5 – 12.8]	[30.3 – 380.2]	[5.6 – 77.0]	[50.1 – 421.0]	[5.2 – 49.0]	[54.1 – 607.6]	[9.4 – 95.7]	[228.9 – 377.9]	[39.4 – 43.6]
TN	[30.9 – 35.5]	[7.9 – 7.9]	[86.5 – 94.1]	[13.9 – 14.3]	[115.0 – 154.4]	[14.0 – 16.3]	[11.8 – 79.0]	[3.3 – 7.8]	[18.7 – 302.4]	[1.3 – 17.0]
TX	[29.1 – 32.7]	[7.0 – 7.0]	[68.3 – 75.3]	[12.0 – 12.3]	[142.7 – 145.9]	[22.5 – 22.6]	[18.8 – 29.7]	[5.5 – 6.2]	[30.2 – 224.5]	[3.9 – 12.0]
UT	[15.9 – 19.7]	[4.7 – 4.7]	[13.9 – 169.1]	[3.5 – 36.0]	[66.1 – 90.5]	[13.2 – 13.9]	[11.5 – 68.9]	[4.9 – 9.1]	[141.0 – 403.8]	[18.0 – 40.0]
VA	[12.5 – 15.4]	[4.9 – 5.0]	[45.0 – 51.4]	[9.3 – 9.7]	[73.9 – 92.0]	[12.4 – 14.1]	[11.6 – 25.7]	[4.7 – 5.7]	[4.5 – 215.4]	[1.5 – 18.6]
WA	[8.1 – 11.1]	[3.5 – 3.6]	[17.7 – 50.9]	[5.3 – 7.6]	[63.0 – 77.1]	[12.9 – 14.2]	[11.1 – 26.6]	[3.8 – 4.5]	[57.2 – 133.9]	[9.1 – 14.6]
WV	[11.8 – 17.3]	[3.6 – 3.7]	[13.9 – 73.6]	[3.0 – 7.0]	[0.23 – 40.9]	[1.1 – 14.2]	[14.2 – 146.7]	[1.1 – 15.5]	[17.3 – 293.2]	[3.1 – 54.5]
WI	[18.7 – 22.7]	[7.4 – 7.4]	[68.8 – 108.0]	[17.2 – 18.1]	[116.8 – 139.7]	[20.1 – 20.9]	[50.9 – 83.1]	[9.7 – 10.7]	[70.5 – 226.3]	[14.6 – 23.3]

Table S4: Conservative 95% interval estimates of the age-adjusted YPLL and mortality rates for NH Whites, NH Blacks, Hispanics, NH Asians, and NH AIAN's in the U.S. and in each examined state with respect to cumulative COVID-19 deaths according to data from the National Center for Health Statistics as of 30 December 2020. The upper reference age used to define YPLL is 75 years.
