# Supplementary material for: Racial and Ethnic Disparities in Years of Potential Life Lost Attributable to COVID-19 in the United States: An Analysis of 45 States and the District of Columbia
Source: Int J Environ Res Public Health. 2021 Mar 12;18(6):2921. doi: 10.3390/ijerph18062921 (PMC8000338; doi:10.3390/ijerph18062921)
Supplement: Supplementary file 1 [file ijerph-18-02921-s001.zip › Supplementary Materials/Table S5.pdf]

State	NH Black		Hispanic		NH Asian		NH AIAN	
	YPLL	Mortality	YPLL	Mortality	YPLL	Mortality	YPLL	Mortality
US	[2.84 – 2.97]	[1.91 – 1.99]	[2.90 – 3.02]	[1.95 – 2.03]	[0.99 – 1.17]	[0.83 – 0.95]	[3.79 – 5.06]	[1.82 – 2.70]
AL	[2.79 – 3.32]	[1.74 – 1.81]	[2.63 – 4.59]	[1.22 – 1.95]	[0.20 – 2.95]	[0.12 – 1.57]	[0.24 – 4.53]	[0.13 – 2.19]
AZ	[1.86 – 5.50]	[1.74 – 2.87]	[4.41 – 6.22]	[3.43 – 3.79]	[0.73 – 4.28]	[0.95 – 2.10]	[14.79 – 23.70]	[7.42 – 8.97]
AR	[2.01 – 2.64]	[1.42 – 1.47]	[2.81 – 4.20]	[1.78 – 1.92]	[0.21 – 2.86]	[0.17 – 1.62]	[0.13 – 2.42]	[0.19 – 1.90]
CA	[3.31 – 3.89]	[2.18 – 2.29]	[6.16 – 6.45]	[3.64 – 3.69]	[1.25 – 1.50]	[1.20 – 1.25]	[1.32 – 5.63]	[1.28 – 2.36]
CO	[2.82 – 6.50]	[2.04 – 2.62]	[4.24 – 6.41]	[2.76 – 2.97]	[1.71 – 6.55]	[1.60 – 2.27]	[4.09 – 22.40]	[1.26 – 5.82]
CT	[3.55 – 6.37]	[2.47 – 2.73]	[2.79 – 4.48]	[1.66 – 1.90]	[0.71 – 3.04]	[0.58 – 1.12]	[0.01 – 23.20]	[0.21 – 7.44]
DE	[0.92 – 4.39]	[1.55 – 1.89]	[0.50 – 6.21]	[0.63 – 2.10]	[0.06 – 2.01]	[0.17 – 1.76]	[0.35 – 13.67]	[0.79 – 10.53]
DC	[3.91 – 10.69]	[3.14 – 3.65]	[8.36 – 24.29]	[4.41 – 5.97]	[0.67 – 16.30]	[0.49 – 4.64]	[1.22 – 56.22]	[0.65 – 10.26]
FL	[3.91 – 4.30]	[2.80 – 2.83]	[2.53 – 2.82]	[2.21 – 2.24]	[0.90 – 1.75]	[0.92 – 1.02]	[0.92 – 10.31]	[0.21 – 1.78]
GA	[2.66 – 3.02]	[1.92 – 1.96]	[3.32 – 4.05]	[1.82 – 1.94]	[0.58 – 1.71]	[0.64 – 0.81]	[0.34 – 4.65]	[0.16 – 2.20]
ID	[0.87 – 21.78]	[0.87 – 12.62]	[2.96 – 6.96]	[2.46 – 2.90]	[0.65 – 12.42]	[0.29 – 3.16]	[0.69 – 14.50]	[0.40 – 3.88]
IL	[4.13 – 5.16]	[2.00 – 2.10]	[5.60 – 6.84]	[2.65 – 2.79]	[1.20 – 2.44]	[1.07 – 1.26]	[0.49 – 20.37]	[0.18 – 5.28]
IN	[2.71 – 3.67]	[1.89 – 1.95]	[2.81 – 3.80]	[1.51 – 1.58]	[0.48 – 2.04]	[0.81 – 1.06]	[0.29 – 11.12]	[0.16 – 2.02]
IA	[2.61 – 5.15]	[1.99 – 2.20]	[3.19 – 4.73]	[1.67 – 1.82]	[0.66 – 3.38]	[1.44 – 1.83]	[2.02 – 25.09]	[0.72 – 8.00]
KS	[2.12 – 7.58]	[1.82 – 2.52]	[3.10 – 6.58]	[2.26 – 2.80]	[0.27 – 7.74]	[0.42 – 2.17]	[2.00 – 31.90]	[1.04 – 6.87]
KY	[2.08 – 3.36]	[1.86 – 2.02]	[3.65 – 5.80]	[1.31 – 2.07]	[0.37 – 4.71]	[0.20 – 1.79]	[0.02 – 4.14]	[0.26 – 4.07]
LA	[3.67 – 4.38]	[2.20 – 2.28]	[1.86 – 3.96]	[1.10 – 1.47]	[0.52 – 2.24]	[0.35 – 1.33]	[0.43 – 7.60]	[0.16 – 2.35]
MD	[3.19 – 4.34]	[2.15 – 2.24]	[7.20 – 9.95]	[2.85 – 3.21]	[0.77 – 2.27]	[0.90 – 1.09]	[0.47 – 13.77]	[0.31 – 4.03]
MA	[3.03 – 4.79]	[1.87 – 2.17]	[2.67 – 3.98]	[1.59 – 1.88]	[0.83 – 2.11]	[0.86 – 1.17]	[0.46 – 30.33]	[0.23 – 7.93]
MI	[5.77 – 6.67]	[3.27 – 3.48]	[3.09 – 4.69]	[1.87 – 2.69]	[0.34 – 2.33]	[0.55 – 1.43]	[0.71 – 8.14]	[0.76 – 4.44]
MN	[4.43 – 6.67]	[2.40 – 2.87]	[4.58 – 8.64]	[2.01 – 2.72]	[3.82 – 6.05]	[1.75 – 2.19]	[3.29 – 13.73]	[1.48 – 3.67]
MS	[2.71 – 3.27]	[1.92 – 1.98]	[1.09 – 3.78]	[0.37 – 1.42]	[0.57 – 4.39]	[0.56 – 2.32]	[17.97 – 34.13]	[5.89 – 9.46]
MO	[2.30 – 3.07]	[1.67 – 1.76]	[2.82 – 3.97]	[1.25 – 1.56]	[0.41 – 2.31]	[0.83 – 1.31]	[0.32 – 5.05]	[0.18 – 2.46]
MT	[3.26 – 53.89]	[1.12 – 26.66]	[1.55 – 12.14]	[1.16 – 3.52]	[0.91 – 17.22]	[0.73 – 6.41]	[11.14 – 22.46]	[6.69 – 7.46]
NE	[1.52 – 7.38]	[1.51 – 2.35]	[3.33 – 7.45]	[2.40 – 3.02]	[0.29 – 6.31]	[0.19 – 2.71]	[0.99 – 20.04]	[0.59 – 7.63]
NV	[2.49 – 4.47]	[2.05 – 2.21]	[3.42 – 5.15]	[2.80 – 2.93]	[1.25 – 2.25]	[1.62 – 1.73]	[1.47 – 15.77]	[0.89 – 3.94]
NH	[1.11 – 54.19]	[1.04 – 11.01]	[0.28 – 21.60]	[0.42 – 4.50]	[0.53 – 25.86]	[0.37 – 4.15]	[0.00 – 57.70]	[0.00 – 4.95]
NJ	[3.70 – 3.99]	[2.20 – 2.23]	[4.44 – 4.76]	[2.36 – 2.39]	[1.30 – 1.78]	[1.13 – 1.17]	[1.00 – 10.92]	[0.25 – 1.81]
NM	[0.48 – 12.28]	[0.35 – 3.06]	[1.43 – 3.91]	[1.79 – 2.04]	[0.12 – 4.29]	[0.28 – 3.24]	[11.83 – 31.63]	[9.84 – 11.31]
NY	[3.84 – 5.76]	[2.77 – 3.32]	[4.57 – 6.49]	[2.94 – 3.52]	[1.91 – 3.80]	[1.55 – 2.28]	[0.24 – 29.90]	[0.29 – 15.33]
NC	[3.16 – 4.05]	[2.31 – 2.42]	[6.57 – 9.71]	[3.07 – 3.53]	[0.69 – 5.41]	[1.09 – 1.82]	[0.71 – 8.36]	[0.99 – 2.61]
ND	[0.31 – 15.37]	[0.45 – 13.61]	[0.95 – 21.94]	[0.48 – 10.16]	[0.01 – 14.99]	[0.51 – 13.59]	[4.14 – 14.58]	[2.51 – 4.91]
OH	[2.43 – 2.95]	[1.55 – 1.59]	[1.95 – 2.77]	[1.05 – 1.20]	[0.66 – 1.91]	[0.79 – 0.99]	[0.47 – 6.19]	[0.17 – 2.06]
OK	[1.12 – 2.53]	[1.25 – 1.37]	[2.12 – 4.06]	[1.78 – 1.94]	[0.41 – 3.34]	[0.66 – 1.43]	[2.25 – 3.69]	[1.83 – 1.91]
OR	[1.55 – 19.85]	[0.47 – 5.04]	[6.57 – 9.66]	[3.56 – 4.39]	[0.57 – 4.25]	[1.34 – 2.33]	[1.74 – 23.82]	[0.58 – 6.03]
PA	[3.89 – 4.61]	[2.34 – 2.42]	[3.50 – 4.29]	[1.97 – 2.14]	[1.30 – 2.45]	[1.24 – 1.45]	[0.01 – 11.72]	[0.16 – 3.31]
RI	[2.08 – 10.31]	[1.63 – 2.17]	[1.55 – 6.27]	[1.52 – 1.81]	[0.67 – 13.03]	[0.22 – 1.84]	[0.01 – 34.71]	[0.39 – 7.28]
SC	[3.35 – 4.25]	[2.13 – 2.19]	[3.10 – 4.25]	[1.16 – 1.62]	[0.26 – 3.22]	[0.32 – 1.49]	[0.62 – 8.89]	[0.32 – 4.16]
SD	[0.73 – 12.36]	[0.43 – 6.14]	[1.22 – 14.13]	[0.40 – 3.90]	[1.36 – 19.89]	[0.73 – 7.58]	[5.61 – 12.40]	[3.08 – 3.48]
TN	[2.44 – 3.02]	[1.75 – 1.81]	[3.25 – 4.96]	[1.77 – 2.07]	[0.31 – 2.54]	[0.42 – 1.00]	[0.52 – 9.63]	[0.16 – 2.17]
TX	[2.10 – 2.57]	[1.70 – 1.76]	[4.38 – 4.99]	[3.19 – 3.24]	[0.58 – 1.01]	[0.78 – 0.89]	[0.93 – 7.66]	[0.55 – 1.72]
UT	[0.72 – 10.38]	[0.75 – 7.76]	[3.42 – 5.58]	[2.80 – 2.99]	[0.60 – 4.23]	[1.03 – 1.96]	[7.31 – 24.85]	[3.81 – 8.58]
VA	[2.94 – 4.08]	[1.88 – 1.98]	[4.84 – 7.34]	[2.49 – 2.87]	[0.75 – 2.03]	[0.94 – 1.16]	[0.27 – 16.92]	[0.30 – 3.81]
WA	[1.62 – 6.11]	[1.45 – 2.13]	[5.77 – 9.34]	[3.56 – 4.00]	[1.03 – 3.21]	[1.03 – 1.27]	[5.24 – 16.17]	[2.52 – 4.12]
WV	[0.86 – 6.05]	[0.81 – 1.95]	[0.01 – 3.36]	[0.29 – 3.93]	[0.84 – 12.14]	[0.31 – 4.28]	[1.04 – 24.02]	[0.83 – 15.22]
WI	[3.06 – 5.71]	[2.32 – 2.46]	[5.19 – 7.38]	[2.71 – 2.84]	[2.27 – 4.39]	[1.31 – 1.46]	[3.15 – 11.94]	[1.96 – 3.16]

Table S5: Conservative 95% interval estimates of the age-adjusted YPLL and mortality rate ratios for NH Blacks, Hispanics, NH Asians, and NH AIAN's relative to NH Whites in the U.S. and in each examined state with respect to cumulative COVID-19 deaths according to data from the National Center for Health Statistics as of 30 December 2020. The upper reference age used to define YPLL is 75 years.
